# Supplementary material for: Complexome profiling of the Chlamydomonas psb28 mutant reveals TEF5 as an early PSII assembly factor
Source: Plant Cell. 2025 Mar 17;37(6):koaf055. doi: 10.1093/plcell/koaf055 (PMC12164590; doi:10.1093/plcell/koaf055)
Supplement: koaf055_Supplementary_Data [file koaf055_supplementary_data.zip › psb28_interactive.zip!/plots/intensity/qualityPlots/peptides/_Cre03.g206450.t1.2.html]

### Cre03.g206450.t1.2

Trivial name:   
SeqCoverage [median]: 45.30  
Mass: 6.76 kDa  
  
 

| Sequence | MissedCleavage | Mass [Da] | wtIntensity | muIntensity | Score |
| --- | --- | --- | --- | --- | --- |
| AEEAAIGGPITSSGGHH | 0 | 1589.74 | 4932.84 | 3453.14 | 134.06 |
| NLATYDAAQR | 0 | 1121.55 | 16398.75 | 15669.23 | 113.66 |
| QKAEEAAIGGPITSSGGHH | 1 | 1845.90 | 8532.60 | 4971.03 | 107.73 |
|  |  |  | 29864.2 | 24093.4 |  |
